# Central Retinal Artery Occlusion with Sparing of Cilioretinal Artery Post Pulmonary Artery Stenting

**DOI:** 10.7759/cureus.2128

**Published:** 2018-01-29

**Authors:** Kok-Tian Oo, Mohd Rizal Mohd-Zain, Ismail Shatriah

**Affiliations:** 1 Department of Ophthalmology, School of Medical Sciences, Universiti Sains Malaysia, 16150 Kubang Kerian, Kelantan, Malaysia; 2 Department of Pediatrics, School of Medical Sciences, Universiti Sains Malaysia, 16150 Kubang Kerian, Kelantan, Malaysia

**Keywords:** pulmonary artery stenosis, pulmonary artey stenting, central retina artery occlusion, pediatric

## Abstract

Central retinal arterial occlusion is an ocular emergency. Central retinal artery occlusion following cardiac procedures have been described in adults. We describe a pediatric patient who developed central retinal artery occlusion following pulmonary artery stenting. It is important to highlight this potential risk to ensure early diagnosis and prompt treatment.

## Introduction

Retinal emboli and retinal artery occlusions are known to occur following greater arterial circulation procedures, including left ventricular assisted device surgery, coronary artery bypass graft surgery, carotid endarterectomy, and carotid artery stenting [1-2]. Pulmonary artery stenting is a rare procedure that may lead to central retinal artery occlusion. We describe a case of severe visual loss in a teenager who developed central retinal artery occlusion following a pulmonary artery stenting procedure.

## Case presentation

A 16-year-old girl with underlying congenital pulmonary atresia and an intact ventricular septum was referred for ophthalmology assessment at day six after a left pulmonary artery stenting procedure. The patient complained of a sudden onset of painless, blurred vision in the right eye after regaining consciousness from the procedure. She had undergone four heart surgeries that included a Blalock-Taussig shunt at two months of age, a bi-directional Glenn shunt at the age of two years, a Fontan procedure at eight years old, and a left pulmonary artery stenting six days prior to consultation.

**Figure 1:**
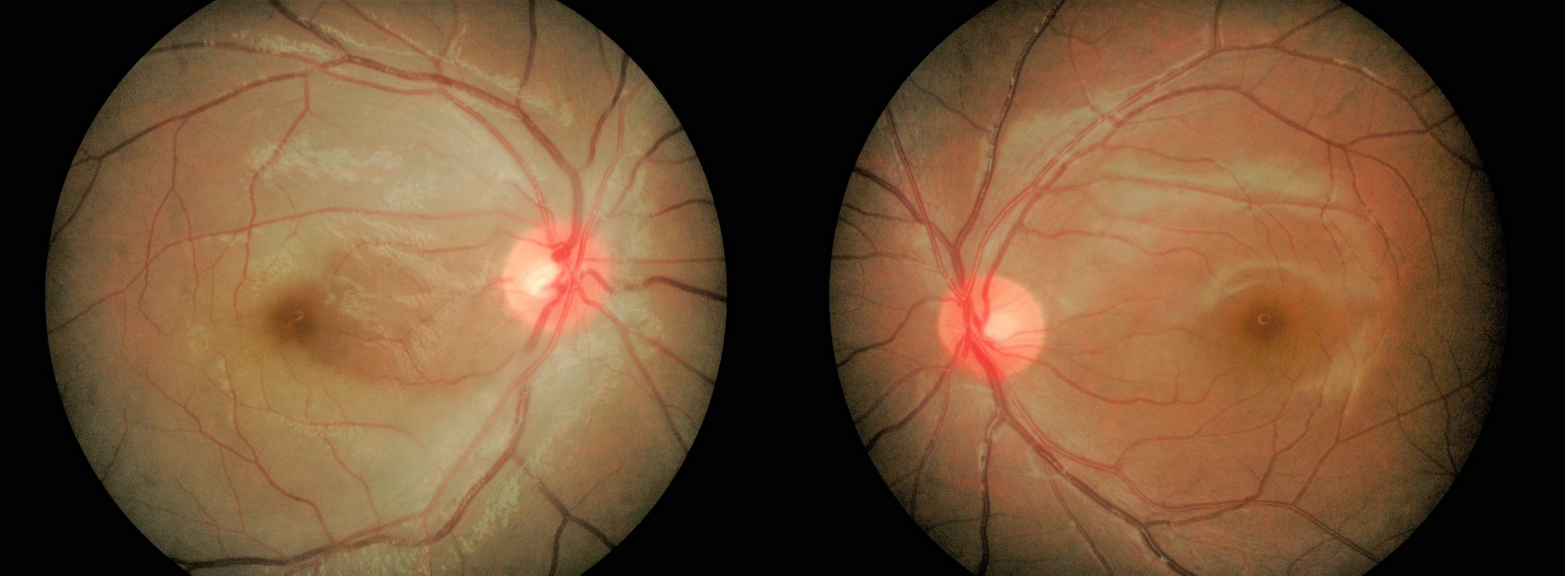
Central retinal artery occlusion with patent cilioretinal artery in the right eye, and normal fundus finding in the fellow eye

Her presenting visual acuity was 20/160 OD and 20/20 OS. There was a marked afferent pupillary defect in the right eye. The intraocular pressures were normal in both eyes. Dilated funduscopic examination showed generalized edematous pale retina with evidence of a sparing papillomacular bundle in the right eye (Figure 1). No obvious embolus was noted. The left eye fundus examination was unremarkable.

**Figure 2:**
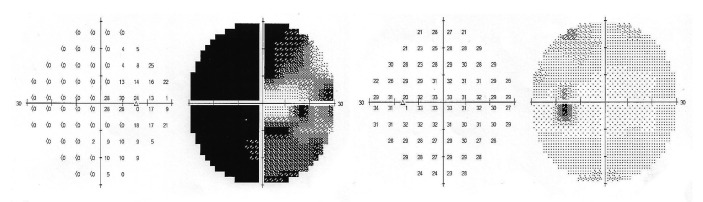
Generalized field loss sparing the central island of vision in the right eye. Normal visual field in the left eyeGeneralized field loss sparing the central island of vision in the right eye. Normal visual field in the left eye

Humphrey visual field demonstrated generalized field loss sparing the central island in the affected eye. The fellow eye was normal (Figure 2). 

She was diagnosed with right central retinal artery occlusion with cilioretinal artery sparing. The patient's parents declined fundus fluorescein angiography.

**Figure 3:**
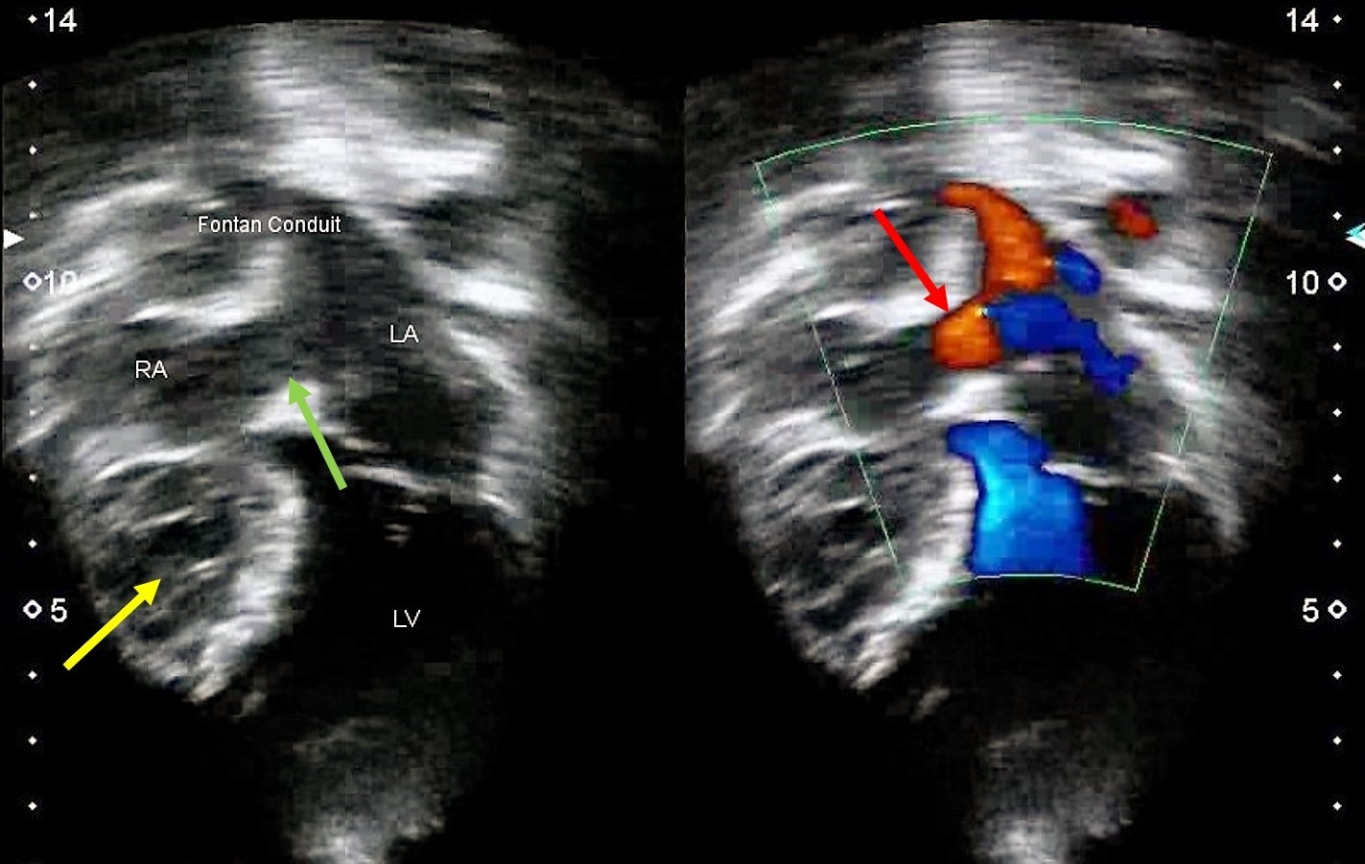
Echocardiogram showing a hypoplastic right ventricle (yellow arrow) and an atrial septal defect (green arrow) with no intracardiac thrombus formation. Color doppler imaging demonstrating blood flowing from the right to the left atrium (red arrow)

The patient was immediately referred to the cardiologist for reassessment. Systemic examination was essentially normal. An urgent echocardiogram revealed an atrial septal defect with no intracardiac thrombus. The color doppler imaging showed blood flow from the right to left atrium via atrial septal defect (Figure 3). 

**Figure 4:**
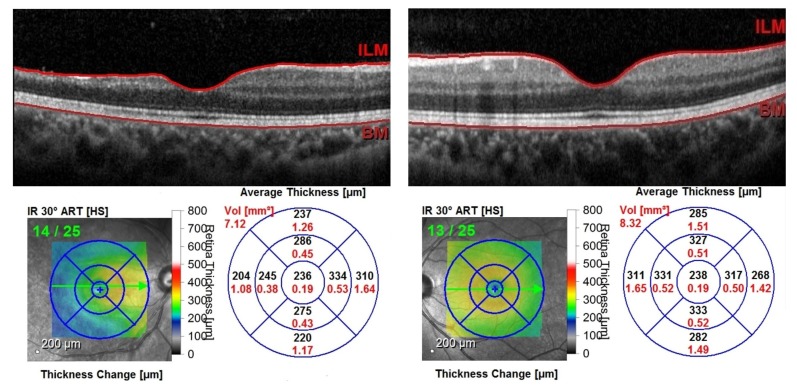
Cross-sectional optical coherence tomography of the right eye showed retinal atrophy of the temporal part of the macula. Normal macula in the left eye

Patient’s previous dosage of oral warfarin was readjusted with the aim of maintaining the international normalized ratio (INR) at 2.5. The patient was reviewed at three months after the incident. The visual acuity showed improvement, with 20/50 OD and 20/20 OS. There was no rubeosis iridis, and intraocular pressure was normal. Optical coherence tomography showed atrophic changes of inner retina layer at the temporal aspect with a preserved papillomacular bundle in the right eye and normal thickness in the left eye (Figure 4). 

She was under close monitoring of her systemic condition and blood INR profile. The latest INR was 2.6. There was no recurrence of central retinal artery occlusion in the affected eye or in the fellow eye, which was also closely observed. No signs of cerebral vascular events were noted during follow-up visits.

## Discussion

Our patient was diagnosed with pulmonary atresia and intact ventricular septum. She underwent three cardiac surgeries in the first eight years of her life, including a right Blalock-Taussig procedure, which is a temporary shunt connecting the right subclavian artery to the right pulmonary artery. A Glenn shunt is the second operation that removes the first shunt and forms a connection from the superior vena cava to the right pulmonary artery. The Fontan procedure is the third or final surgery that connects the inferior vena cava to the pulmonary artery.

The goal of the Fontan procedure is to divert the systemic venous return directly to the pulmonary arteries. Thus, it reduces volume overload of the single ventricle and increases systemic oxygenation. Despite the advantages, this procedure carries risk of right atrial dilatation, which can lead to additional problems, such as increased thrombus formation, arrhythmias, and pulmonary vein compression. It has been reported that venous thromboembolism occurs in between 3–16% of patients, and stroke or arterial thrombi occurs in 3–19% of patients after Fontan procedures [3].

The left pulmonary artery stent procedure is designed to improve the diameter of the left pulmonary artery. This is designed to improve the caliber and flow of blood to the left pulmonary artery, enhancing the blood flow of Fontan circulation to the lungs [4]. We postulated that the procedure may cause mobilization of micro-thrombi into the superior vena cava and right atrium. Subsequently, the thrombi likely gained access to the left atrium and hence the systemic and retinal circulation via the atrial septal defect. This explains the clinical manifestation of central retinal artery occlusion in our patient. There was no sign of cerebral thrombosis in our patient during close follow-up visits.

Duration of time from the onset of visual impairment is critical in the management of central retinal artery occlusion. This is because neuronal death and retinal infarction evolve progressively in a time-dependent fashion determined by both the duration and severity of the ischemic insult [5]. Ocular massage, carbogen therapy, and anterior chamber paracentesis performed more than 24 hours after an attack of central retinal artery occlusion do not facilitate visual improvement [6-7]. This explains why our patient did not receive any active management from the ophthalmology team because the onset of retinal ischemia had exceeded the 24 hours period.

Management of central retinal artery is also aimed at preventing subsequent vascular events, including stroke, myocardial infarction, and cardiovascular death [8]. Walker and Gatzoulis (2005) reported that thrombi are detected most commonly in the first year after a Fontan surgery, with a plateau after 3.5 years, and followed by a second peak after 10 years. The late peak in risk would be of particular concern in the case of long-term survivors following Fontan surgery [9]. Therefore, anticoagulant treatment and cautious INR monitoring are crucial.

## Conclusions

Central retinal artery occlusion following cardiac procedures is rare and difficult to predict. Risk and preoperative explanation need to be emphasized. Early diagnosis may help to prevent permanent visual loss.
